# Bio fabrication of silver nanoparticles with antibacterial and cytotoxic abilities using lichens

**DOI:** 10.1038/s41598-020-73683-z

**Published:** 2020-10-08

**Authors:** Mona A. Alqahtani, Monerah R. Al Othman, Afrah E. Mohammed

**Affiliations:** 1 Department of Biology and Microbiology, Faculty of Science, King Saud University, P.O. Box 22452, Riyadh, 11495 Saudi Arabia; 2 Department of Biology, College of Science, Princess Nourah Bint Abdulrahman University, P.O. Box 84428, Riyadh, 11671 Saudi Arabia

**Keywords:** Microbiology, Antimicrobials, Antimicrobial resistance

## Abstract

Recently, increase bacterial resistance to antimicrobial compounds issue constitutes a real threat to human health. One of the useful materials for bacterial control is Silver nanoparticles (AgNPs). Researchers tend to use biogenic agents to synthesize stable and safe AgNPs. The principal aim of this study was to investigate the ability of lichen in AgNPs formation and to find out their suppression ability to MDR bacteria as well as their cytotoxic activity. In the current study, lichens (*Xanthoria parietina*, *Flavopunctelia flaventior*) were collected from the south of the Kingdom of Saudi Arabia. Lichens methanolic extracts were used for conversion of Ag ions to AgNPs. Prepared biogenic AgNPs were characterized by Ultraviolet–Visible (UV–Vis) Spectroscopy, Transmission electron microscopy (TEM), Dynamic Light Scattering (DLS) and Zeta potential and Energy-Dispersive X-ray Spectroscopy (EDS). Lichens Secondary metabolites were determined by Fourier-Transform Infrared Spectroscopy (FTIR) and Gas Chromatography–Mass Spectrometry (GC–MS). The antibacterial activity and synergistic effect of AgNPs were evaluated against pathogenic bacteria, including gram-positive; Methicillin-resistant *Staphylococcus aureus* (MRSA), Vancomycin-resistant *Enterococcus* (VRE), and gram-negative; (*Pseudomonas aeruginosa, Escherichia coli*) as well as the reference strains (ATCC) using the agar disk diffusion method. Cytotoxic effect of biogenic AgNPs was tested against HCT 116 (Human Colorectal Cancer cell), MDA-MB-231 (Breast cancer cell), and FaDu (Pharynx cancer cell) by MTT test. TEM imaging showed well-dispersed spherical particles of 1–40 nm size as well as zeta size showed 69–145 nm. Furthermore, FTIR and GC–MS identified various lichen chemical molecules. On the other hand, the highest antibacterial activity of AgNPs was noticed against *P. aeruginosa*, followed by MRSA, VRE, and *E. coli*. AgNPs influence on gram-negative bacteria was greater than that on gram-positive bacteria and their synergistic effect with some antibiotics was noted against examined microbes. Moreover, higher cytotoxicity for biogenic AgNPs against FaDu and HCT 116 cell line in relation to MDA-MB-231 was noted. Given the current findings, the biogenic AgNPs mediated by lichens had positive antibacterial, synergistic and cytotoxic powers. Therefore, they might be considered as a promising candidate to combat the multi-drug resistance organisms and some cancer cells.

## Introduction

Recently, development of bacterial resistance to antibiotics and related issues constitutes a real threat to human health. In view of public health, new antimicrobial compounds with varied range of activities have to be improved and developed to minimize the resistance of bacteria^1^. The World Health Organization (WHO) has issued a universal antimicrobial resistance map, giving alert that the world will soon suffer from a ‘post-antibiotic’ phenomenon. Recently, resistant bacteria for drugs showed great concern because they developed quickly and spread around the world according to (WHO) report^2^; consequently, there is an urgent need to develop alternatives. In the strictest sense, organism resist multi antimicrobial compounds in vitro is defined as Multi-Drug Resistant (MDR) organisms^3^. Antibiotic resistance is considered as one of the strongest potential factors in severely infected patients together with the virulence of pathogen resulting in sickness and mortality^4^. Both bacterial types (Gram-positive and Gram-negative) assumed to exhibit resistance to antimicrobial agents. However, gram-negative bacteria with multi-drug resistant ability require special attention. Since such a problem grows continually therefore, searching for solutions and recommendations for proper microbial treatment are needed^5,6^. Excessive uptake of antibiotics might lead to adaptational resistance and using of wide-spectrum agents could assist in extending the resistance cycle. These factors have participated in the development of resistance as well as the outbreak of multi-drug resistant organisms^7,8^. The gradual emergence of bacterial resistance poses a hazard to public health therefore, it is essential to be investigated in atrial to find out reasonable solution. Silver is one of the most important metals that inhibits the growth of microbes; it has been used in ancient times for therapeutic purposes. The high reactivity of silver ions for protein binding, could be the main reason for changes appear in the structure of cell wall and membrane of bacteria resulting in cell death^9^. Using this metal in its natural state has adverse effects on the human body. The human body absorbs silver and silver compounds by ingestion, inhalation, or exposure through the skin or mucous membranes and deposited in small amounts in the kidneys and liver^10^. Therefore, converting silver to another form might be required. One of the useful materials for infection control is Silver nanoparticles (AgNPs) which are characterized by small size, a property which offers them unique physicochemical features that differ from their bulk materials, which is basically attributed to greater ratio between their surface area and volume^11^. Generally, nanotechnology is a base of novel applications such as nanomaterials, nanometrology, electronics, optoelectronics, nanobiotechnology and industrial applications^12,13^. In particularly, AgNPs have been used as delivery tool in gene and colon cancer therapies^14,15^ as well as antibacterial activities^16–18^. Therefore, AgNPs could be a promising approach to treat MDR because it is rarely to find microbes that resist AgNPs since microbes need various mutation to develop such phenomenon^19^ because of metals several targets in microbe^20^. Recently, researchers tend to use biogenic agents such as plant extracts to synthesize stable AgNPs by treating the aqueous solution of AgNO_3_ with the plant extracts as reducing agents^21^. Plant and plant-derived materials are rich in secondary metabolic substances like polysaccharides, vitamins, and proteins. Therefore, it might be the right choice for the biosynthesis of metal nanoparticles^22^. In the current study, different lichen types were used as biogenic mediators for nanoparticles formation. Lichens are a symbiotic self-sustaining group formed by fungi and algae that have been utilized in the form of medicines, diet and feed, perfumes, spices, and for a variety of purposes^10^. Lichens medical application depends on its containing uniquely different active biological substances; to date, about 1050 bioactive ingredients were recognized^23^. Lichen active compounds showed diversified types of biological actions containing antioxidant, antimicrobial, cytotoxic, phytotoxic, wound healing, enzyme inhibitory, antiherbivore, analgesic, anti-termite, anti-inflammatory, and others^24^. Researchers used different types of lichens for AgNPs formation as [*Parmeliopsis ambigua*,* Punctelia subrudecta*,* Evernia mesomorpha*,* Xanthoparmelia plitti*^25^, *Parmotrema praesorediosum*^26^, *Cetraria islandica*^27^, *Ramalina dumeticola*^28^, *Usnea longissima*^17^, *Parmelia perlata*^29^, *Parmotrema tinctorum*^30^, and *Cladonia rangiferina*^31^]. However, information about *Xanthoria parietina* and *Flavopunctelia flaventior* usage in AgNPs formation is lacking. Therefore, the aim of the current study was to report such species for the first time as bio mediators in AgNPs formation. In the current study, selected lichen types were collected from the south of the Kingdom of Saudi Arabia and applied as mediator for the conversion of Ag ions to AgNPs. Biogenic AgNPs were characterized using UV–Vis Spectroscopy, TEM, DLS, Zeta potential, and EDS. Lichens Secondary metabolites were determined by FTIR and GC–MS. The antibacterial activity of AgNPs and their synergistic potential with antibiotics were evaluated against pathogenic bacteria, including gram-positive; (MRSA, VRE) and gram-negative; (*P. aeruginosa*,* E. coli*) as well as ATCC of the same bacteria using the agar disk diffusion method. Cytotoxic effect of biogenic AgNPs was tested against HCT 116 (Human Colorectal Cancer cell), MDA-MB-231 (Breast cancer cell), and FaDu (Pharynx cancer cell) by MTT assay.

## Materials and methods

### Materials

Silver nitrate was purchased from Saudi Overseas Marketing and Trading Company (SOMATCO), Riyadh, Saudi Arabia. For the antibacterial assays, Blood agar, Mueller–Hinton Agar (MHA), and Mueller–Hinton Broth (MHB) was purchased from Saudi Prepared Media Laboratory (SPML) Company, Riyadh, Saudi Arabia. All the clinical isolates and American Type Culture Collection (ATCC) bacteria were obtained from Microbiology Laboratory in King Faisal Specialist Hospital & Research Centre (KFSH&RC), Riyadh, Saudi Arabia. Antibiotic discs were from OXOID; Tetracycline 30 µg for *S. aureus*, Linezolid 30 µg for *E. faecium*, Gentamicin 10 µg for *P. aeruginosa* and Ampicillin 10 µg for *E. coli*. Such appropriate antibiotics for ATCC bacteria were selected by antibiotic disc quality control laboratory reports.

### Lichens collection

**Figure 1:**
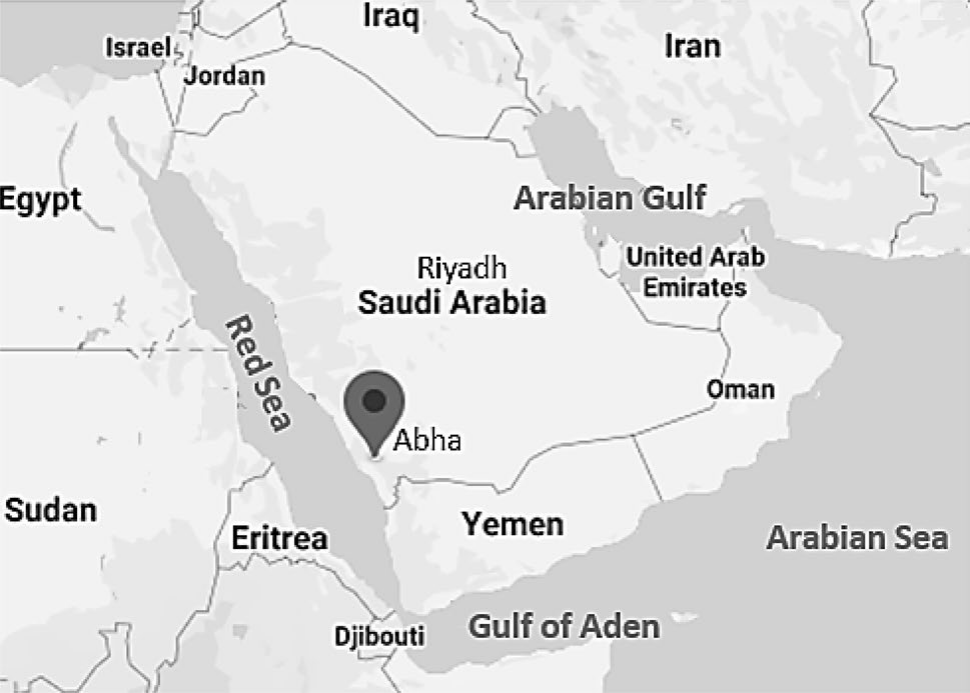
The location of collected lichen.

**Figure 2:**
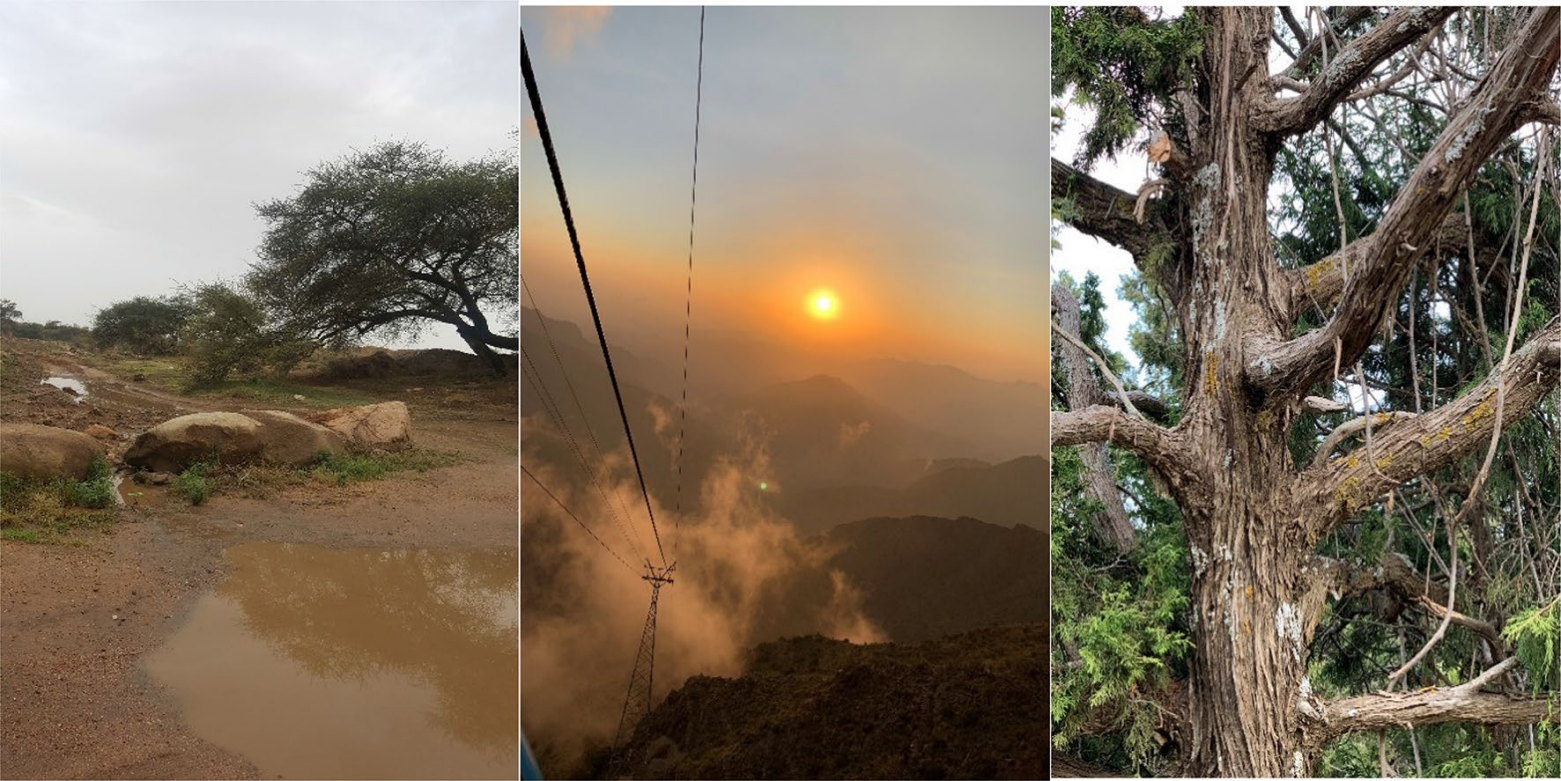
Habitat of collected lichens.

**Figure 3:**
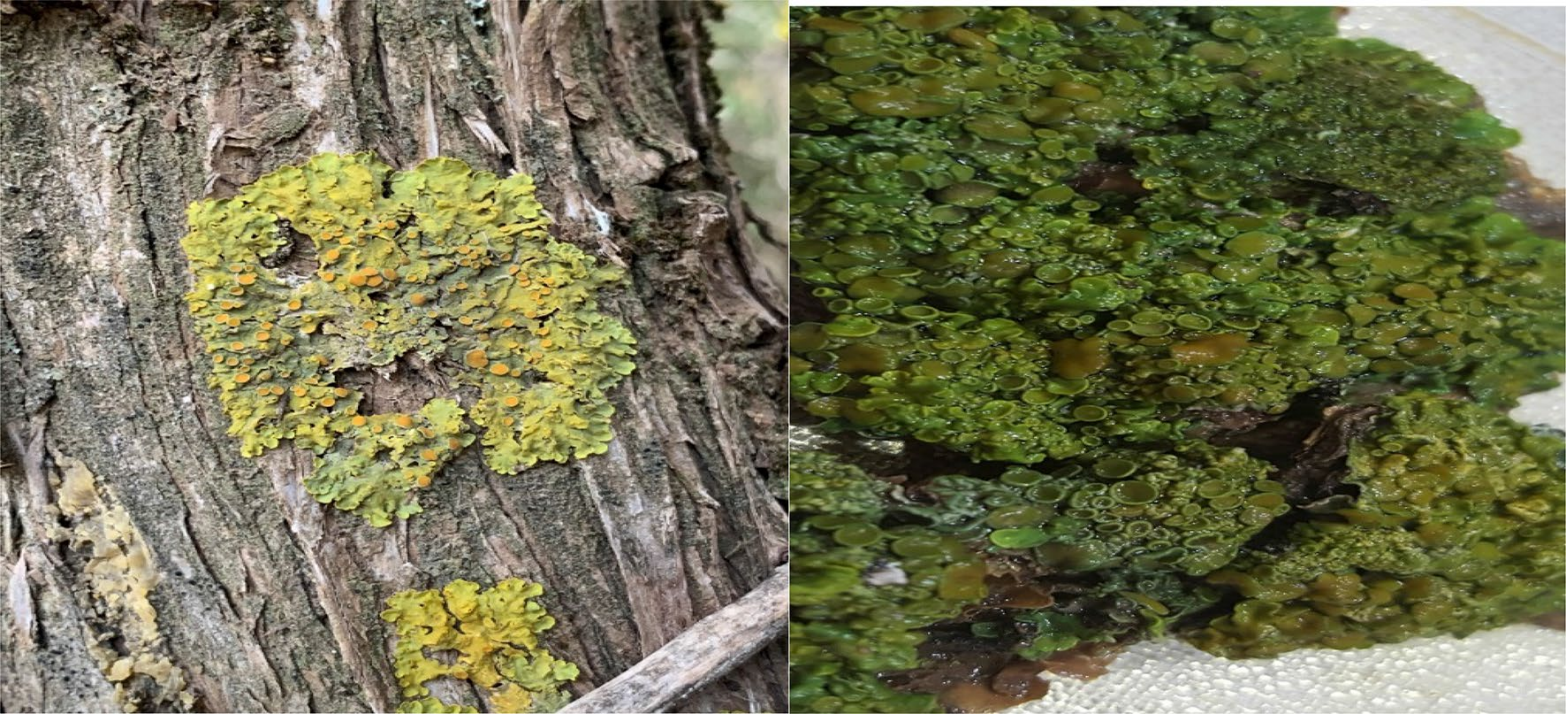
Lichen type *Xanthoria parietina* on the tree (left) and after cleaning (right).

**Figure 4:**
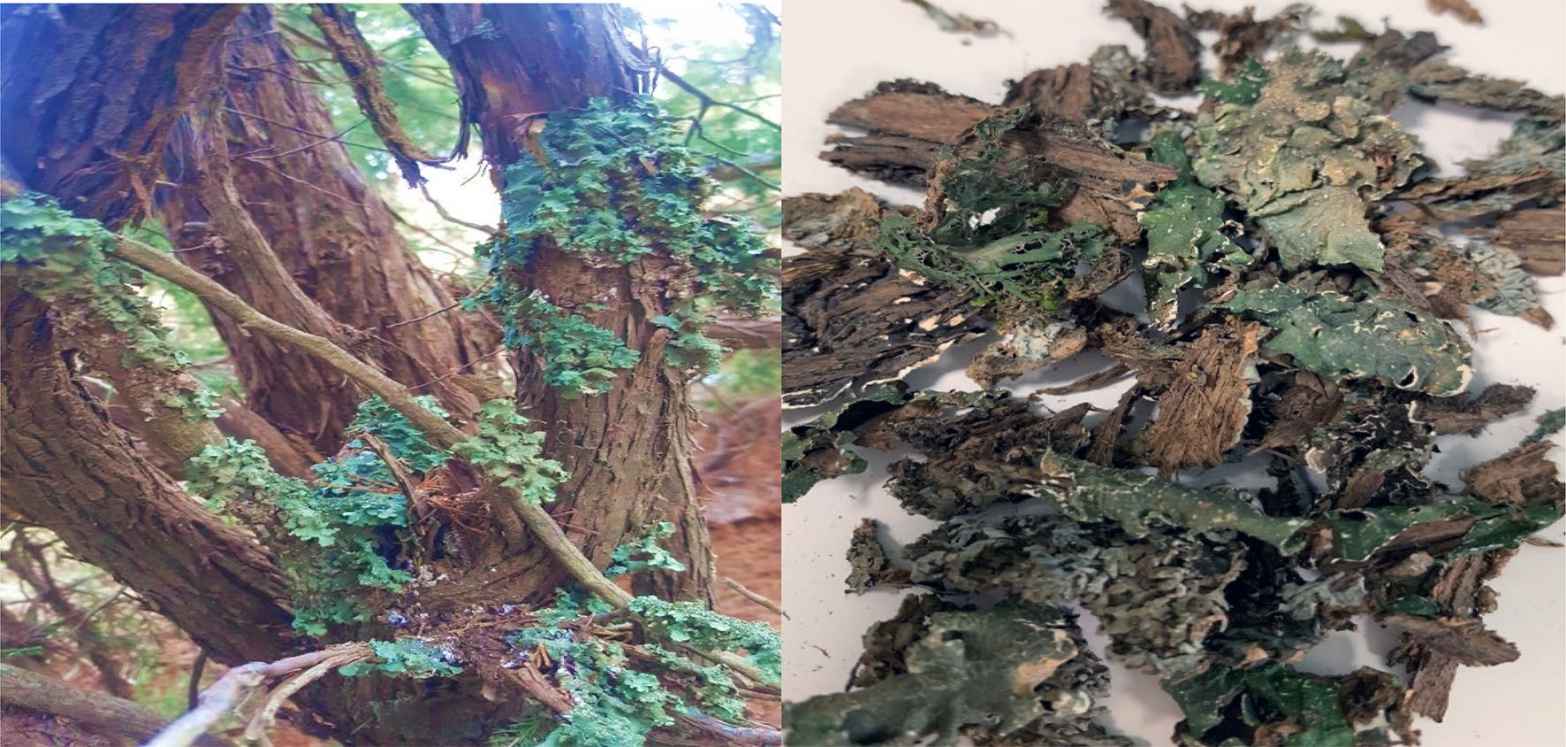
Lichen type *Flavopunctelia flaventior* on the tree (left) and after cleaning (right).

Lichens samples were collected from Al-Soudah and Bani Mazin Which are mountainous areas at an altitude of about 2900 m of Abha city, South of Saudi Arabia in august 2017, 2018 (Fig. 1). Samples collected at temperature of 19 °C, rainy weather, and foggy is clear in (Fig. 2). During samples collection, some points were taken into consideration, growth form, thallus intact and the margins visibility. Two lichen samples were collected from the trees, thereafter, samples were purified and segregated according to their growth forms and the type of fruiting bodies [Apothecia, Perithecia, Sterile -stretched apothecia lirellae-]^32^ as shown in Figs. 3, 4. The lichens were washed using distilled water to remove surface impurities and extraneous materials. Then lichen samples were air-dried at room temperature for three days then ground by a grinder to a fine powder. The samples were kept in containers for further usage.

## Methods

### Identification of lichen samples

Lichen type *Xanthoria parietina *‘*Xa*’ and type *Flavopunctelia flaventior *‘*Ff*’ were identified by morphological, anatomical characters, and chemical tests. The spot test was carried out by direct application of the reagents such as Reagent 10% potassium hydroxide (K), Reagent calcium hypochlorite (C), Reagent (KC), Reagent (Pd) "Steiner's solution"^32^. Identified samples are presented in Fig. 3 for (Xa) and in Fig. 4 for (Ff), both samples were early found and documented in Saudi Arabia^33^.

### Extraction of lichens

An alcohol extract of each lichen was prepared by mixing 10 g of a lichen powder with 100 mL of 80% methanol. The mixtures were left shaking (160 rpm for 24 h) at room temperature. Then mixture was filtered through 'Whatman filter paper Grade 1’ and further filtered through 'Whatman filter paper Grade 3’ for more extract purification then kept at 4 °C. Furthermore, 100 mL methanol was added to the rest of the extract and left more 24 h on the shaker under the same previous conditions. The mixture was filtered twice in the same way as before and the methanol was removed by a rotary evaporator device. The round bottom flask was weighed before and after extraction, then the difference was calculated, the concentration of the final extract was 10 mg/mL. The extract was closed tightly and saved at 4 °C until used^34^.

### Optimization of AgNPs synthesis conditions

The parameters that may affect the nanoparticles biosynthesis were tested such as the ratio of lichen extract to AgNO_3_ solution was tested according to (1:9, 1:3, 1:2, 1:1), AgNO_3_ concentration (1 mM, 10 mM), incubation time (24 h, 48 h, 72 h) and temperature degree (25 °C; 40 °C). After the color change, the particle size was measured by zetasizer. Thereafter, AgNPs were synthesized by application of the conditions that provided small particle size^27^ and considered as an optimum conditions. AgNPs prepared using *Xanthoria parietina* is known as Xa-AgNPs and those prepared using *Flavopunctelia flaventior* are Ff-AgNPs.

### Synthesis of AgNPs

For the synthesis of the AgNPs, about 5 mL of the methanol extract of lichen (10 mg/mL) were mixed with 15 mL of AgNO_3_ (10 mM) solution in a flask and shaken for 3:30 h under dark conditions then allowed to react at 40 °C for 72 h^27^.

### Characterization of biogenic AgNPs

Different methods for the characterization of the biogenic AgNPs prepared in the current study were used for nanoparticles description such as:

#### Ultraviolet–Visible Spectroscopy

Ultraviolet–Visible (UV–Vis) Spectroscopy absorption was measured using a spectrophotometer (BIOCHROM Libra S60PC, Serial Number: 119377, England). All measurements were performed for the mixture after 24 h of reaction within the range of 300–600 nm and deionized water was used as a blank^25^.

#### Transmission electron microscopy (TEM)

The size distribution and morphology of AgNPs were investigated at 80 kV voltage by TEM (JEM-1011, JEOL, Japan). samples were prepared by drop-coating on carbon-coated (200 mesh) TEM grids^26^.

#### Dynamic Light Scattering (DLS) and Zeta potential

The size distribution pattern was evaluated by a dynamic light scattering technique and the electrical charge of particles by zeta potential were the measurement with a Zetasizer (NANO ZSP, Malvern Instruments Ltd, Serial Number: MAL1118778, ver 7.11, UK) according to Siddiqi et al.^17^.

#### Energy-dispersive X-ray spectroscopy (EDS)

EDS was used for the elemental analysis and confirmed the presence of the silver element precisely using SEM (JEOL, JED-2200 series, Japan)^35^.

#### Fourier-transform infrared spectroscopy (FTIR)

FTIR measurements were carried out to identify the potential biomolecules in lichen extract responsible for reducing and capping the reduced AgNPs. The spectra were recorded on FTIR spectroscopy (SPECTRUM100, Perkin-Elmer, USA) using a diffuse reflectance accessory, and the scanning data were obtained with a range between 450–3500 cm^−1^^27^.

### Identification of Lichens Secondary metabolites by (GC–MS)

The Gas chromatography-mass spectrometry (GC–MS) analyses of lichens methanol extracts were conducted by using (AGILENT Technologies 220 Ion Trap GC/MS, USA). Helium was used as the carrier gas with column (Flow rate 1 mL/min; Pressure 8.2317 psi; Average Velocity 36.623 cm/s; Holdup Flow 1.3653 min; Post run 0.99996 mL/min; 450 °C: 30 m × 250 μm × 0.25 μm). The injector and interface were operated at 250 °C; Initial oven temperature was 70 °C to finally programmed to 250 °C with run time is at 52 min. The compounds of lichen extracts were analyzed using the National Institute of Standards and Technology (NIST) chemical database^36,37^.

### Evaluation of antibacterial activity of AgNPs

#### Antibacterial susceptibility testing (AST)

The antibacterial activity of AgNPs was evaluated against four pathogenic bacteria including two gram-positive (MRSA, VRE), and two gram-negative (*P. aeruginosa*, *E. coli*) as well as the reference strains using the agar disk diffusion method. Pure cultures of each strain were sub-cultured on blood agar plates and grown for 24 h at 37 °C. By direct colony suspension method, McFarland standard 0.5 bacterial suspensions (1.5 × 10^8^ CFU/mL) in the saline tube was prepared using McFarland reader. MHA plates were inoculated by tested strains using a petri plate rotator. The sterile discs were saturated by 20 µL of AgNPs and kept for drying under aseptic conditions. Then dried discs were transported to bacterial cultured agar surface using sterile forceps with pressure for the discs to be closely bonded. Sterile distilled water was used as negative control and antibiotic susceptibility discs were used as positive controls. After 15 min of discs application, plates were inverted and incubated at 35 °C for 16–18 h according to the Clinical and Laboratory Standards Institute^38^. The zone of inhibition around the discs was measured by Vernier caliper. Antibacterial activity was investigated and zone diameter breakpoints (mm) for antibiotics were determined according to M02 and M07 from^39,40^. Such mentioned methods were also applied for lichen methanol extracts and methanol was used as positive control^26^.

#### MICs and MBCs determination

The minimal inhibitory concentrations (MICs) of the AgNPs were determined by the microdilution method in 96-well microtiter plates. The obtained concentration range of lichen extract was from 2.5 to 0.0098 mg/mL. Positive control (media contains inoculum with antibiotic), negative control (media contain inoculum), and AgNPs solution (media with AgNPs) was applied in the last three columns. All plates were incubated for 18–20 h at 35 °C. MICs were determined by comparing to positive and negative control wells. The lowest concentration with no growth (turbidity or pellet) was defined as MICs. Results expressed as the mean values of two independent replicates. MICs breakpoints (μg/mL) for antibiotics was determined by CLSI^39,40^. The minimum bactericidal concentrations (MBCs) was be determined from broth microdilution by sub-culturing a sample from wells on MHA plates by loop 1 μL. After 24 h of plates incubation, the concentration that kills 99.9% of bacterial growth has been defined as MBC^41^. Furthermore, the tolerance level was calculated as MBCs/MICs ratios to find-out the expected action for AgNPs (bactericidal or bacteriostatic) against tested bacteria^16^.

#### Synergistic effect of AgNPs and antibiotics against MDR pathogens

The disk diffusion method was used to test the effect of antibiotics in combination with AgNPs against the MDR bacteria. Test plates were inoculated with the microbes in the same way as AST. AgNPs at concentration of 20 μg was added to each disc of Tetracycline (TE), Linezolid (LZD), Gentamicin (CN), and Ampicillin (AMP), then transported to bacterial cultured agar to test the prepared discs activities. Antibiotic discs were used as positive control. The plates were incubated for 18 h at 35 °C^42^ then the inhibition zone around discs were assessed.

### Cytotoxicity of AgNPs

#### Cell lines

HCT 116, MDA-MB-231 and FaDu were used (Table 1). All cells were grown in GIBCO Dulbecco’s modified Eagle’s medium (DMEM) which containing 10% fetal bovine serum (FBC), 2 mM glutamine, 100 U penicillin and 0.1 mg/mL streptomycin. Culture flasks were incubated for 4 days at 37 °C, 99–100% humidity in 5% CO_2_ incubator. The subculture of cells was performed daily to keep the cells from over confluent. After four days of incubation, the media was removed from cell culture flasks T-25 using a sterile pipette. The cells were washed by 5 mL phosphate buffer saline (PBS) for 1 min then added to 1 mL of trypsin. After 2 min, culture flask was checked under the microscope for cells detachment from substrate. 2 mL of media was added to the flask to stop the reaction because the FBS inactivated the trypsin. The cells were placed in tubes and centrifuged for 5 min 6000 rpm to dispose of the trypsin. 2 mL of the medium was put for precipitation then the number of cells was counted^43^.

**Table 1:** Cancer cell lines information.

Cell line	Tissue	Disease
HCT 116	Colon	Colorectal carcinoma
MDA-MB-231	Mammary gland/breast	Adenocarcinoma
FaDu	Pharynx	Squamous cell carcinoma

Organism: human; morphology: epithelial; culture properties: adherent.

#### Cells count

A ratio of 1:2 cell culture contains 50 µL and 50 µL of Trypan Blue Solution (0.4%) in Eppendorf tube was added and injected in cell counting slide at rate of 20 µL for each side. The slide was entered in (LUNA automated cell counter; Logos Biosystems, Gyunggi-do, Korea). Serial dilutions of cells in media were prepared to obtain cell count (5 × 10^4^) cells/mL^43,44^.

#### Application of AgNPs on cell culture (Microplate 96-well)

100 µL of media in (Dilution plate) was added for each well except first and last row due to its exposure to air more than the internal rows. 50 µL of Xa-AgNPs was placed in the first well of the second and third row. 50 µL of lichen extract (Xa) was placed in the first well of the fourth and fifth row. 50 µL of methanol was placed in the first well of the sixth and seventh row and methanol was used as control. Using serial dilution method by multichannel micropipette; 50 µL was transferred from the first column to the second column and so on to last column and disposal of the rest 50 µL. 120 µL of cell suspension in cultivation plate was added to each well except first and last row. 60 µL of each column in dilution plate was transferred to the same column in cultivation plate; More precisely 60 µL of (tested material with media) was transferred from the last column of dilution plate to last column of cultivation plate (from lowest concentration to the highest concentration), and so on with all columns. These methods were applied to Ff and Ff-AgNPs and the experiment was performed in duplicate^35^.

#### MTT assay

250 mg of MTT 3-(4,5-dimethylthiazol-2-yl)-2,5-diphenyl tetrazolium bromide was dissolved in 50 mL of PBS and stirred with a magnetic stirrer for homogenization. 20 µL of MTT was added to each well then incubated for 2 h at 37 °C, 99–100% humidity in 5% CO_2_ incubator. The medium was removed by suction. Cells were washed with PBS and centrifuged to removed dead cells and cellular debris. 100 µL of isopropanol (C_3_H_8_O) was added to each well then was shaken for 10 min. MTT conversion to purple-colored formazan crystals is due to presence of viable cells with active metabolism. Absorbances were measured at 595 nm by ELISA reader (ANTHOS 2010 Microplate Reader, Biochrom LTD, UK). The cell viability was calculated using following formula:

$$ {\text{Cell}}\,{\text{viability}}\,{1}00\% = \, \left( {{\text{OD}}\,{\text{Sample}}/{\text{OD}}\,{\text{Control}}} \right) \, \times {1}00. $$

Half-maximal inhibitory concentration (IC_50_) values represent the concentration of tested materials that required for 50% inhibition of cells growth. IC_50_ values were measured from the regression curves^45^.

### Statistical analysis

Means and standard deviations for antibacterial activities were calculated using MICROSOFT EXCEL 2019. AgNPs images were chosen as one of the triplicates. ORIGIN software version 6.1 (ORIGIN Lab Corporation, Northampton, USA) statistical analyses was used for MTT assay for IC_50_ assessment.

## Results

### Biosynthesis of AgNPs

**Figure 5:**
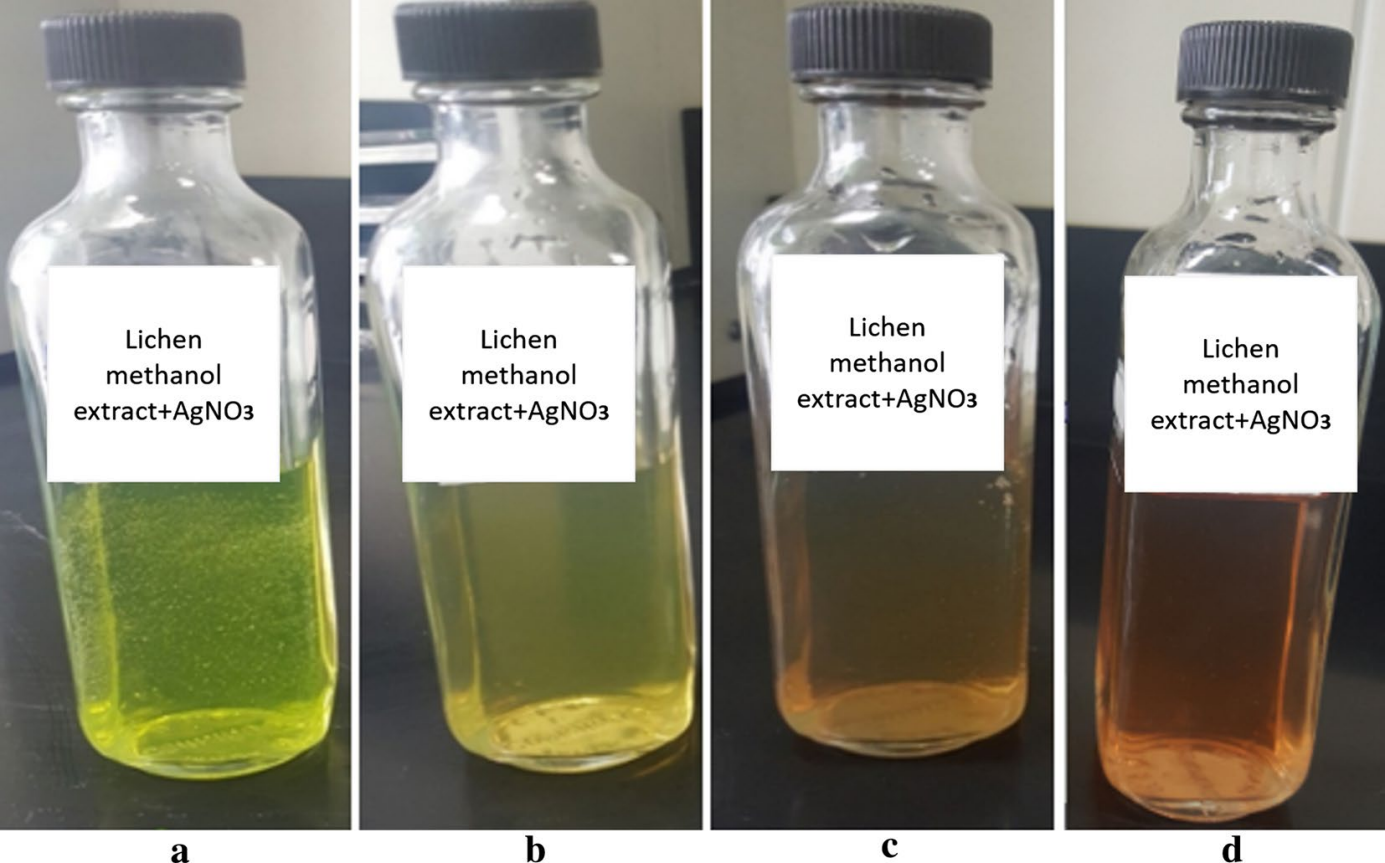
Color change during the reaction time for during Xa-AgNPs preparation, where (**a**) is indicating the colour immediately after mixing, (**b**) after 24 h, (**c**) after 48 h and (**d**) is the colour after 72 h.

To obtain the best AgNPs size, conditions were optimized and adjusted as follows: the two lichen types were extracted by methanol and each extract was added to AgNO_3_ (10 mM) at a ratio of 1:3 at 40 °C for 72 h. It was noticed that increasing the reaction temperature and AgNO_3_ concentration reduced the reaction time since conversion time of Ag ions to AgNPs at 40 °C for AgNO_3_ at 10 mM concentration was shorter when compared to that at 25 °C and 1 mM. The reaction mixture immediately assumed yellow green colour for Xa-AgNPs (Fig. 5) which was then started to change gradually after 24 h in time dependant manner. After 72 h, the mixture was turned to brown and no more colour changes were noted. Such colour stability might point to attainment of maximum reduction time but also it revealed that, colour intensity was highly time dependant. both Xa-AgNPs and Ff-AgNPs assume similar pattern in colour development and stable nanomaterials for more than 4 months were approved.

### Characterization of biogenic AgNPs

**Figure 6:**
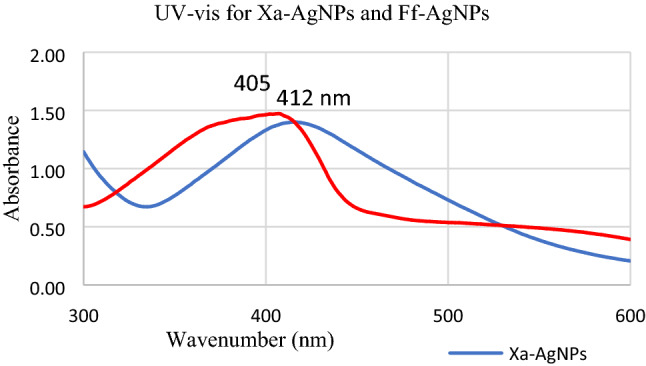
UV–Vis for Xa-AgNPs and Ff-AgNPs.

**Figure 7:**
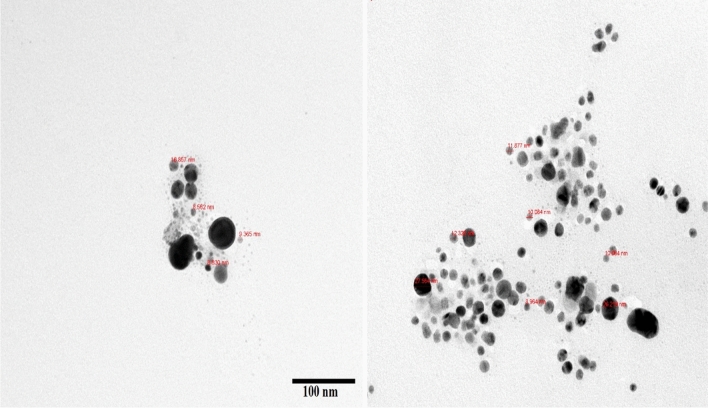
TEM images for Xa-AgNPs (left) and Ff-AgNPs (right).

**Figure 8:**
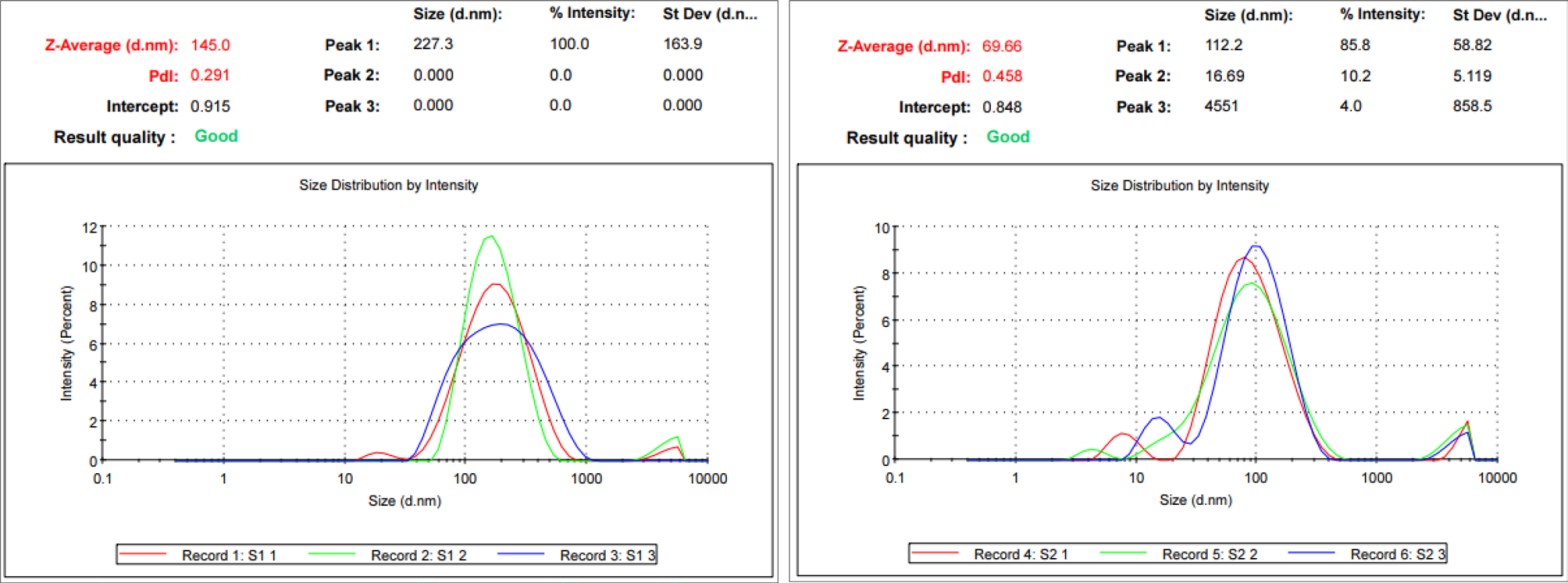
Particles size of Xa-AgNPs (left), Ff-AgNPs (right).

**Figure 9:**
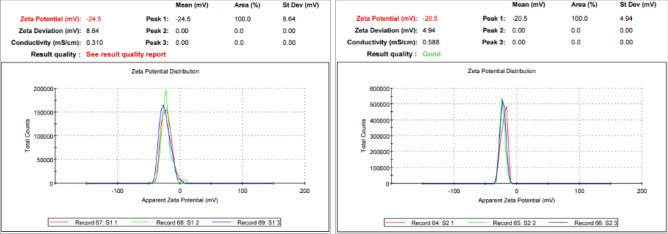
Zeta Potential of Xa-AgNPs (left), Ff-AgNPs (right).

**Figure 10:**
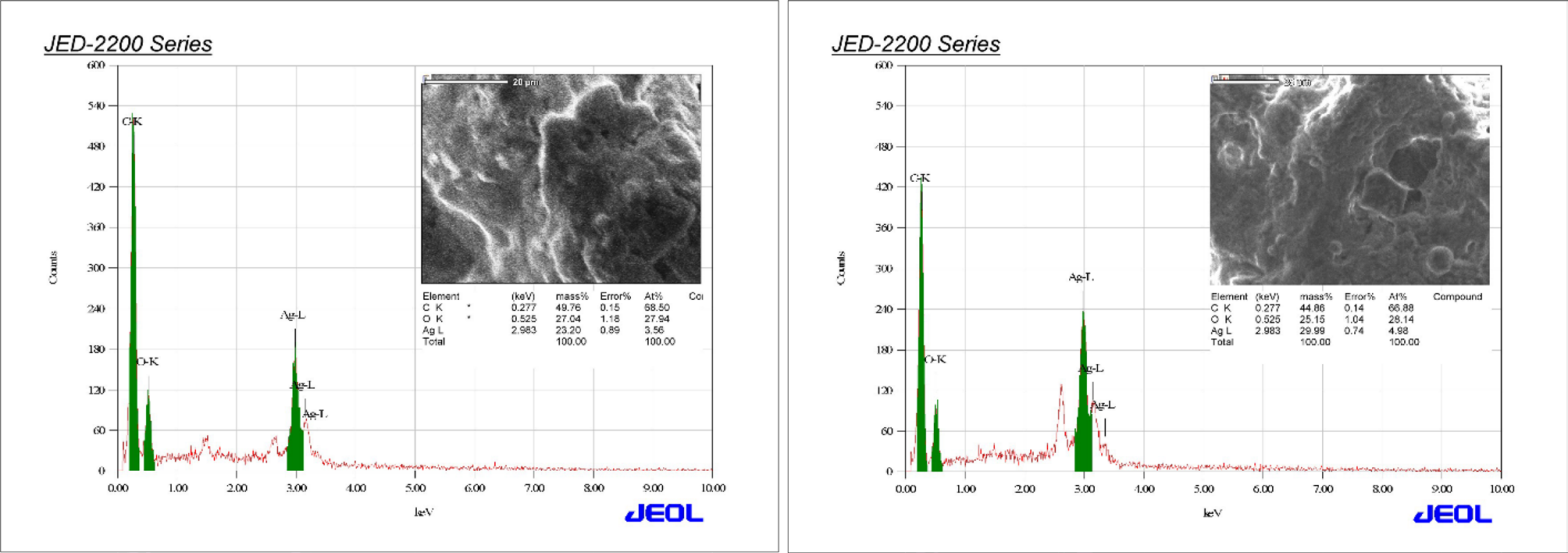
EDS for Xa-AgNPs (left), Ff-AgNPs (right).

**Figure 11:**
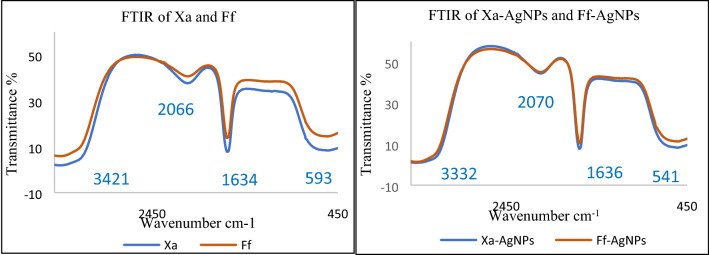
FTIR for lichen extracts Xa and Ff (left) and that for Xa-AgNPs and Ff-AgNPs (right).

Reactions between silver ions and both lichen extracts were monitored by UV–Vis Spectroscopy of AgNPs (Fig. 6). The analysis of UV–Vis spectroscopy showed an appearance of surface plasmon resonance peak at 412 and 405 nm for Xa-AgNPs and Ff-AgNPs respectively. TEM imaging for Xa-AgNPs and Ff-AgNPs showed well-dispersed spherical particles of 1–40 nm size, furthermore, clear capping agents around AgNPs as light colour were detected (Fig. 7). Results obtained from DLS for Xa-AgNPs and Ff-AgNPs had average diameters of 145 and 69 nm with a polydispersity index (PDI) of 0.291 and 0.458, respectively (Fig. 8). Zeta Potential values were − 24, − 20 mV (Fig. 9) for Xa-AgNPs and Ff-AgNPs respectively. Analysis through EDS confirmed the presence of the silver element in both AgNPs beside the carbon and oxygen. The results showed strong silver signals Ag-L at 3 keV, along with carbon peak and oxygen peak (Fig. 10). FTIR spectra characterized the lichen extracts from ‘Xa and Ff’ as well as after AgNPs prepared by their aid (Fig. 11). Absorbance peaks for both lichen types were at 3421 cm^−1^, 2066 cm^−1^, 1634 cm^−1^ and 593 cm^−1^. Additionally, the absorbance peaks for Xa-AgNPs and Ff-AgNPs were at 3421–3332 cm^−1^, 2070 cm^−1^, 1637 cm^−1^, and 541 cm^−1^, representing the role of various functional groups in the bio-reduction of AgNO_3_.

### Identification of Lichens secondary metabolites by (GC–MS)

To find out the chemical constituents that could be involved in conversion of silver ions to AgNPs, Gas chromatography–mass spectrometry was used for lichen methanol extracts. GC–MS analysis of both lichen types showed the presence of 25 compounds (Table 2). Benzenamine, 4-methoxy-*N*-methyl-compound was noticed for both lichen types.

**Table 2:** GC–MS analysis of lichen *Xanthoria parietina *‘*Xa*’* and Flavopunctelia flaventior* ‘Ff’.

Name	Formula	Molecular weight	Retention time	Found in lichen	Nature of compounds
Benzyl bromide	C_7_H_7_BrO	171	0.713	Xa	Benzyl Halide
2-Amino-2-ethyl-1,3-propanediol	C_5_H_13_NO_2_	119	2.279	Xa	Alkanolamine
Dichloran	C_6_H_4_Cl_2_N_2_O_2_	206	26.187	Xa	Nitroaniline
Hexachlorophene	C_13_H_6_Cl_6_O_2_	406	1.480	Xa	Phenolic
Pyrrolidine, 1-methyl-	C_5_H_11_N	85	1.742	Xa	Alkaloid
undecanal	C_11_H_22_O	170	7.2653	Xa	Fatty aldehyde
Benzenamine, 4-methoxy-*N*-methyl-	C_8_H_11_NO	137	13.500 13.515	Xa, Ff	Amine -Aniline
2,5-Cyclohexadiene-1,4-dione, 2,6-bis(1,1-dimethylethyl)-	C_14_H_20_O_2_	220	26.172	Ff	Benzoquinone
2′,5′-Dihydroxypropiophenone	C_9_H_10_O_3_	166	22.039	Ff	Phenolic
3,4-Dimethyl-2-hexanone	C_8_H_16_O	128	7.245	Ff	Ketone
Acetaldehyde, methylhydrazone	C_3_H_8_N_2_	72	31.704	Ff	Methyl-hydrazine
Atropine	C_17_H_23_NO_3_	289	14.386	Ff	Alkaloid
Beclomethasone	C_22_H_29_ClO_5_	408	1.493	Ff	Corticosteroid
Butanenitrile 3-methyl-	C_5_H_9_N	83	1.847	Ff	Nitrile
Colchicine	C_22_H_25_NO_6_	399	37.123	Ff	Alkaloid
Cortisone	C_21_H_28_NO_5_	360	44.087	Ff	Corticosteroid
Cyclopentadecanol	C_15_H_30_O	226	24.798	Ff	Alcoholic
Cyclopentanone, oxime	C_5_H_9_NO	99	2.277	Ff	Hydroxyl-amine
Dehydrocholic acid	C_24_H_34_O_5_	402	34.141	Ff	Cholagogue and Choleretic
Hydrocortisone acetate	C_23_H_32_O_6_	404	33.827	Ff	Steroid
Isobutane	C_4_H_10_	58	1.710	Ff	Alkane
Octadecanamine	C_18_H_39_N	269	36.685	Ff	Amine
Oxacyclododecan-2-one	C_11_H_20_O_2_	184	7.262	Ff	Fatty ester
Piperidine	C_5_H_11_N	85	1.737	Ff	Alkaloid
Ricinoleic acid	C_18_H_34_O_3_	298	21.246	Ff	Fatty acyls—octadecanoid

### Evaluation of antibacterial activity of AgNPs

**Figure 12:**
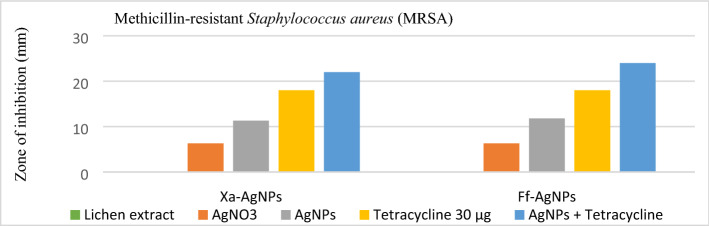
Synergistic effect of Xa-AgNPs and Ff-AgNPs and antibiotics on MRSA.

The antibacterial activities of lichen extracts and the biogenic AgNPs were evaluated against MDR and ATCC bacterial strains. No antibacterial activity was detected for lichen extracts however, AgNPs inhibited the growth of both tested gram-negative and gram-positive strains (Table 3). The highest antibacterial activity of Xa-AgNPs and Ff-AgNPs was noticed against *P. aeruginosa* followed by MRSA, VRE and *E. coli*, respectively. No significant variations were observed in the activity of Xa-AgNPs and Ff-AgNPs against tested ATCC and MDR bacteria. MRSA, VRE, *P. aeruginosa* MDR, and *E. coli* ATCC were more sensitive of Ff-AgNPs compared to Xa-AgNPs. Furthermore, the MICs and MBCs of Xa-AgNPs and Ff-AgNPs against tested bacteria were listed in Table 4. The MICs (Lack of turbidity or pellet in the test tube) was observed up to the concentration of 0.019 and 0.039 mg/mL for both biogenic AgNPs against *P. aeruginosa* (MDR and ATCC), while MBCs were recorded at a concentration of 0.078 and 0.039 mg/mL for Xa-AgNPs and Ff-AgNPs, respectively against *P. aeruginosa* MDR. However same MBC was noticed for both AgNPs against *P. aeruginosa* ATCC and *S. aureus* ATCC (Table 4). For tolerance level of AgNPs against tested bacteria, MBCs/MICs ratios were calculated and determined whether AgNPs action were bacteriostatic or bactericidal. Xa-AgNPs and Ff-AgNPs showed bactericidal effect against all tested bacterial strains since the tolerance levels were ≤ 4 (Table 5). In addition, the possible synergistic effect was noted against all tested strains when combination between each of Xa-AgNPs and Ff-AgNPs with antibiotics was done. Results showed clear inhibition zone around the discs for all tested bacterial strains (Fig. 12). Such combination of each Xa-AgNPs and Ff-AgNPs and antibiotics provided efficient activity against MRSA and VRE more than *P. aeruginosa* and *E. coli* (Table 6). Ff-AgNPs increased the efficiency antibiotic TE up to 133% against MRSA, while its activity increased up to 123% against VER when combined with each prepared AgNPs.

**Table 3:** Agar disk diffusion assay of Xa-AgNPs and Ff-AgNPs.

The diameter of the zone of inhibition of AgNPs (mm) at (5 mg/mL)				
Bacteria strains	Xa-AgNPs^a^ that size 145	Ff-AgNPs^a^ that size 69	AgNO_3_	(+) Control ‘Antibiotics’
MRSA	11.3 ± 0.2	11.8 ± 0.2	6.3 ± 0.5	TE30: 18 ± 0
*S. aureus* ATCC	12.3 ± 0.2	11.6 ± 0.5	6.8 ± 0	TE30: 21 ± 0
VRE	10.1 ± 0.2	10.3 ± 0.2	5.1 ± 0.2	LZD30: 21 ± 0
*E. faecium* ATCC	10.1 ± 0.2	10.3 ± 0.2	5 ± 0.2	LZD30: 24 ± 0
*P. aeruginosa* MDR	13 ± 0	13.5 ± 0.5	6.3 ± 0.2	CN10: 11 ± 0
*P. aeruginosa* ATCC	12.8 ± 0.2	12.5 ± 0.5	6.6 ± 0.5	CN10:18 ± 0
*E. coli* MDR	7.6 ± 0.2	7.5 ± 0.5	4.5 ± 0	AMP10: 10 ± 0
*E. coli* ATCC	7.3 ± 0.5	8.8 ± 0.2	4.3 ± 0.2	AMP10: 20 ± 0

^a^Xa-AgNPs are the AgNPs prepared using *Xanthoria parietina* and Ff-AgNPs are the AgNPs prepared using *Flavopunctelia flaventior*, Methicillin-resistant *Staphylococcus aureus* (MRSA), Vancomycin-resistant *Enterococcus* (VRE), American Type Culture Collection (ATCC), Multi-Drug Resistant (MDR), Tetracycline 30 µg (TE30), Linezolid 30 µg (LZD30), Gentamicin 10 µg (CN10), Ampicillin 10 µg (Amp10). The mean and standard deviation of triplicate were presented in all results.

**Table 4:** MICs and MBCs for the biogenic AgNPs.

Bacteria strains	MICs (mg/mL)		MBCs (mg/mL)	
	Xa-AgNPs^a^	Ff-AgNPs^a^	Xa-AgNPs^a^	Ff-AgNPs^a^
MRSA	0.078	0.156	0.312	0.312
*S. aureus* ATCC	0.078	0.039	0.156	0.078
VRE	0.156	0.078	0.625	0.312
*E. faecium* ATCC	0.156	0.078	0.625	0.156
*P. aeruginosa* MDR	0.039	0.019	0.078	0.039
*P. aeruginosa* ATCC	0.039	0.019	0.156	0.078
*E. coli* MDR	0.156	0.078	0.312	0.156
*E. coli* ATCC	0.078	0.039	0.312	0.078

^a^Xa-AgNPs are the AgNPs prepared using *Xanthoria parietina* and Ff-AgNPs are the AgNPs prepared using *Flavopunctelia flaventior*.

**Table 5:** Tolerance level (MBC/MIC) of Xa-AgNPs and Ff-AgNPs.

Bacteria strains	Xa-AgNPs^a^	Ff-AgNPs^a^
MRSA	4	2
*S. aureus* ATCC	2	2
VRE	4	4
*E. faecium* ATCC	4	2
*P. aeruginosa* MDR	2	2
*P. aeruginosa* ATCC	4	4
*E. coli* MDR	2	2
*E. coli* ATCC	4	2

^a^Xa-AgNPs are the AgNPs prepared using *Xanthoria parietina* and Ff-AgNPs are the AgNPs prepared using *Flavopunctelia flaventior*.

**Table 6:** Synergistic effect of Xa-AgNPs and Ff-AgNPs and antibiotics against bacteria strains.

Diameter of the zone of inhibition of Xa-AgNPs and Ff-AgNPs (5 mg/mL) with antibiotics (mm)			
Bacteria strains	Xa-AgNPs^a^ + Antibiotic	Ff-AgNPs^a^ + Antibiotic	(+) Control ‘Antibiotics’
MRSA	22 ± 0	24 ± 0	TE 30: 18 ± 0
*S. aureus* ATCC	21.5 ± 0.5	22 ± 0	TE30: 21 ± 0
VRE	26 ± 0	26 ± 0	LZD30: 21 ± 0
*E. faecium* ATCC	25.1 ± 0.2	27 ± 0	LZD30: 22 ± 0
*P. aeruginosa* MDR	13 ± 0	13.5 ± 0	CN10: 11 ± 0
*P. aeruginosa* ATCC	20 ± 0	20 ± 0	CN10:18 ± 0
*E. coli* MDR	11 ± 0	11 ± 0	Amp10: 10 ± 0
*E. coli* ATCC	21 ± 0	20.6 ± 0.5	Amp10: 20 ± 0

^a^Xa-AgNPs are the AgNPs prepared using *Xanthoria parietina* and Ff-AgNPs are the AgNPs prepared using *Flavopunctelia flaventior*.

### Cytotoxicity of AgNPs

Xa-AgNPs and Ff-AgNPs showed higher cytotoxic effect compared to that of lichen extracts ‘Xa and Ff’ (Table 7). Results from the current study indicated higher cytotoxicity for Xa-AgNPs and Ff-AgNPs against FaDu and HCT 116 cell line in relation to MDA-MB-231. Higher efficiency was observed for Ff-AgNPs compared to Xa-AgNPs.

**Table 7:** Cytotoxicity of Xa-AgNPs and Ff-AgNPs against Cancer cell lines.

IC_50_ values µg/mL as measured in MTT assay				
Cancer cell lines	Xa-AgNPs^a^ (145 nm)	Xa-Extract	Ff-AgNPs^a^ (69 nm)	Ff-Extract
MDA-MB-231	250	330	35	40
FaDu	93	170	23	34
HCT 116	96	210	29	38

^a^Xa-AgNPs are the AgNPs prepared using *Xanthoria parietina* and Ff-AgNPs are the AgNPs prepared using *Flavopunctelia flaventior*.

## Discussions

### Bioynthesis of AgNPs

The present study could be considered as the first report indicating the biosynthesis of AgNPs by the aid of the extracts of lichen types *Xanthoria parietina* and *Flavopunctelia flaventior* as well as reporting their antibacterial and cytotoxic activities. Interestingly, methanol has the capability of dissolving polar compounds with a polarity index of 5.1. while water has a polarity index of about 10.2^29^. Our previous study presented the formation of AgNPs by water extract of lichen *Parmotrema clavuliferum*^46^. However, currently methanol was used for extraction of lichens. Optimizing the conditions for fabrication of AgNPs using both lichens tested were confirmed by color change thereafter, particles size average were measured by zetasizer. Factors affected the synthesis of nanoparticles were the ratio of AgNO_3_ to lichen extract, AgNO_3_ concentration, temperature and reaction time^27^. Biogenic AgNPs using *Cetraria islandica* formed in few minutes^27^, 30 min using *Parmelia perlata*^29^, 24 h using *Parmotrema praesorediosum*^26^ and 72 h using *Cladonia rangiferina*^31^. It could be concluded that, variations in time needed for nanoparticle formation might likely be related to solvent type, method and conditions of extraction, secondary metabolites of lichens and their concentration. Our findings were in accordance with the findings of many researchers who reported 1:3 as the ratio of the extract to the silver nitrate, however, they studied aqueous extract of lichens^26,28–31,35^. Siddiqi, et al.^47^ confirmed that, color intensity of the mixture increased with temperature increment and quick formation of nanoparticles was obtained. The first sign for AgNPs synthesis using lichen extracts was the change of color from yellow-green to dark-brown in the reaction mixture. Such conversion could be a clear indication for the reduction of silver ions to AgNPs which is related to surface plasmon resonance (SPR) phenomenon^27^. The exact mechanism included in the process of AgNPs formation using biogenic agents was not clearly known, however, several hypotheses of AgNPs formation by the green synthesis was explored in the biological system. The main hypothesis was that, AgNPs are formed underneath the surface of the cell wall and reduced in the presence of biomolecules or enzymes, while intracellular synthesis occurs inside the cells^17,48^. It has been suggested that the silver ions require nicotinamide adenine dinucleotide (NADH) and NADH-dependent nitrate reductase enzymes^49^. In the case of lichens such enzyme is secreted by the fungal partner extracellularly^25^ and several secondary metabolites which act as reducing agents to produce AgNPs from AgNO_3_ without producing toxic by-product^28,30^.

### Characterization of biogenic AgNPs

The SPR peaks for Xa-AgNPs and Ff-AgNPs corresponded to AgNPs production where AgNPs absorb radiation intensely at a wavelength of 400–450 nm due to the transition of electrons^25,26^. The peaks at 400 nm are supposed to be as indicators for spherical shapes of the particles^17^. TEM analysis confirmed the formation of silver nanostructures and visualized of synthesized AgNPs at 100 nm scales. The morphology and size distribution of both Xa-AgNPs and Ff-AgNPs showed spherical particles with nonspecific distribution. Our TEM findings are well matching with reports in the literature where same size range and spherical particles shape were noted^17,26–28,30,31^. It was noticeable that the edges of the particles were lighter than the centres, suggesting that bioorganic molecules such as proteins in lichen and other metabolites capped the AgNPs contributed to reduction of Ag ions to AgNPs^30^. Particle sizes in DLS and Zeta Potential analysis of Xa-AgNPs and Ff-AgNPs with Polydispersity Index (PDI) were in agreement with the particle size early mentioned^17,30^. Zeta Potential provided high negative value of zeta potential for both AgNPs confirming the repulsion among the particles, and the negative value also indicates that nanoparticles have high degrees of stability^50^. The negative potential values could also be due to the presence of bio-organic components in the lichen extract that acted as capping agents. Generally, AgNPs synthesized using biological materials has good mono-dispersity as well as stability^51^. The particle size differed in TEM and DLS because the principles of the techniques in both analyses were different. The particles size in DLS was larger than those detected by TEM micrographs which could be due to the presence of impurities of bio-active molecules of lichen on the AgNPs surface. In addition to above mentioned points, DLS mainly measures the hydrodynamic radius of the nanoparticles^30^. The EDS results are consistent with previous studies that reported a strong peak for Xa-AgNPs and Ff-AgNPs at (3 keV), which is typical for the absorption of metallic silver nano-crystallites. Two impurity peaks were detected below 1 keV, which corresponded to carbon peak (CK) and oxygen peak (OK), that might be originated from the lichen extract^26,28,35^. It is well documented that, lichen extracts contains several metabolites, including antranorin as (+)-praesorediosic acid, and (+)-protopraesorediosic acid that has important roles in the synthesis of AgNPs^28^. The functional groups of Xa and Ff lichen extracts responsible for the reduction of Ag^+^ from AgNO_3_ and stabilization of AgNPs was studied by FTIR^30^. FTIR is an analytical technique to identify organic and inorganic materials that used to obtain an infrared spectrum of absorption or emission of a solid, liquid and gas^29^. The spectrum was observed at 3300–3500 cm^−1^, indicating the presence of polyphenolic –OH group and N–H stretching of amine^17,25^. On the other hand, it was observed that the ratio between the intensity of the bands at 2066–2070 cm^−1^ could be attributed to the –C=C– stretch; alkynes^52^. Intense absorption bands in FTIR at 1600–1650 cm^−1^ might be attributed to amide I due to carbonyl stretch in proteins C=O stretch^25,27,29,30^. A comparison between the spectra of lichen extracts and AgNPs displayed little alterations in the position and the magnitude of the absorption bands indicating using the lichen secondary metabolites in nanoparticle formation.

### Identification of Lichens secondary metabolites by (GC–MS)

The efficiency of lichens might be ascribed to a unique chemo-diversity having secondary metabolites 80% more than those produced by other organisms^29^. In addition, lichens have distinctive chemical compounds that is totally different from those produced by fungi, algae and plants^53^. At least 1050 different compounds have been identified from lichen species. Such compounds may act as antibacterial, antifungal, antiseptic, anti-inflammatory, antioxidant, antiviral, anticancer and antiproliferative agents as well as healing properties has been also confirmed. However, a major obstacle of lichen metabolites to be incorporated into medical applications is the natural toxicity of some secondary metabolites^54^. In Saudi Arabia, a large number of lichen species are present however, they were not well identified which could be related to the complexity of lichen cultivation. GC–MS results are illustrated in Table 2 displaying similarity with reported literature that detected phenols, amines, aldehydes and ketones, besides many other compounds that could be responsible for the reduction and stabilization of AgNPs^17,25,30,31^. Lichens of same species may identified different chemical compounds which could be related to substrate types and concentrations that used for lichen extraction^54^. Compared to previous researches, the chemical compounds detected in both lichen types tested in the current study were [undecanal^55,56^, Piperidine ^29^, and Colchicine^57^]. Benzenamine, 4-methoxy-*N*-methyl-compound was observed in both lichen types of Xa and Ff, it is a form of amine group that possibly contribute in reducing silver ions to AgNPs^58^. Some identified compounds from Xa in the current study had antibacterial activity such as (Benzyl bromide^59^, Hexachlorophene^60^, Pyrrolidine, 1-methyl-^61^, 2-Amino-2-ethyl-1,3-propanediol^62^, undecanal^63^. The compounds that has antibacterial activity in Ff lichen are Oxacyclododecan-2-one^64^, Piperidine^65^, Atropine^66^, 2′,5′-Dihydroxypropiophenone^67,68^, and Colchicine^69^. Furthermore, it has also been demonstrated a chemical compound with antioxidant activity in lichen type Xa such as undecanal^63^. On the other hand, Oxacyclododecan-2-one^70^, Piperidine^65^, Ricinoleic acid^71^, 2′,5′-Dihydroxypropiophenone^68^, 2,5-Cyclohexadiene-1,4-dione, 2,6-bis(1,1-dimethylethyl)^72^, and Colchicine^69^ were identified from lichen type Ff and Dichloran^73^, Hexachlorophene^74^ were found in lichen type Xa, all were characterized by their antifungal activities. Some compounds identified in lichen type Ff showed therapeutic activities such as Beclomethasone that used to treatment of persistent asthma^75^, Colchicine used to treatment of gout^76^, Atropine used to treatment of Arrhythmias^76^ Ff include Beclomethasone^75^ that has anti-inflammatory activity and Octadecylamine^77^ involved with other compounds in materials manufacture that used in drug delivery.

### Evaluation of antibacterial activity of AgNPs

Biogenic AgNPs by the aid of both lichens were examined in the current study for antibacterial activity against different bacteria, however, no antibacterial activity was detected for lichen extracts alone; suggesting that 10 mg/mL concentration was not enough for antibacterial efficacy^26,28–30^. Some investigations reported the antibacterial activity of AgNPs prepared using lichen extracts influenced gram-negative bacteria more than gram-positive bacteria^26,30,31^; however, our study didn’t show the same pattern which is in accordance with some other investigations^17,28,35^. Noticed activity of the AgNPs against *Pseudomonas aeruginosa*; might be due to the cell wall structure of gram-negative^31^ since MRSA and VRE showed less sensitivity. Penetration of the AgNPs in gram-negative bacteria could be much easier than that in gram-positive due to the cell wall that composed of a thinner layer of peptidoglycan compared to gram-positive^28^. Ff-AgNPs showed more efficacy against some bacteria strains (MRSA, VRE, *P. aeruginosa* MDR, *E. coli* ATCC) compared to Xa-AgNPs which might be due to the size of the particles that was 69 nm for Ff-AgNPs, while that for Xa-AgNPs was 145 nm. The chemical components were identified from Ff lichen type were more than those identified in Xa which could also be a reason for high antibacterial activity since some of had antibacterial and antioxidant activities. Recent studies have also reported similar trend of observations regarding antibacterial activity of AgNPs against *P. aeruginosa* in relation to *S. aureus* and *E. coli*^30,31^. *E. coli* in the current study showed less sensitivity to biogenic AgNPs which might be related to the fact that, such antibacterial agents are bacterial specific therefore, AgNPs mechanism against bacteria should be further investigated since no special trend was observed regarding microbial species. It has been noticed that the higher concentration of biogenic AgNPs, the greater the antibacterial activity was detected^30^. Also, smaller AgNPs with a large surface area possesses antibacterial effects greater than that of larger AgNPs size because the smaller particles can easily anchor to bacterial cell wall and penetrate^28^. However, several studies proposed that AgNPs mode of action against bacteria could be related to following points (a) interact with the membrane surface during cell wall synthesis (b) suppression during protein synthesis (c) disruption of transcription process (d) perturbation of metabolic pathways where interact of Ag with the thiol groups of respiratory enzymes of bacteria^17,30^. Furthermore, MICs and MBCs of biogenic AgNPs exhibited good bactericidal activity at low concentrations against tested bacterial strains^78^. No available reports for MICs and MBCs for AgNPs prepared by lichens. The present investigation is in consistence with the other study that revealed the tolerance level was bactericidal for biogenic AgNPs prepared by some plant extracts^16^. Synergistic effect of antibiotics and AgNPs was examined and their combination increased the activity against all tested bacterial strains^79^. Tetracycline, linezolid and gentamicin are protein synthesis inhibitors antibiotics. Tetracycline and gentamicin inhibit protein synthesis by binding the 30S subunit of bacterial ribosome, whereas linezolid binds to 50S subunit. Ampicillin is part of beta-lactam antibiotics group and considered as cell wall synthesis inhibitor^80^. Synergistic results represented a significant consensus with previously findings reported the AgNPs using biogenic extracts^80–85^. Such activity could be of interest to overcome the antibiotic resistant microbes since combination increased the antibiotic activity suggesting easy penetration of the antibiotic active ingredients by the aid of the nanoparticles. Such enhancement of antibiotic activity could be related to the fact that, AgNPs facilitate good transfer to the antibiotic and bind it to the interaction site with the microbe^86^. Same trend of observation was also recorded in various studies^87^.

### Cytotoxicity of AgNPs

To investigate the biogenic AgNPs cytotoxicity, MTT test was done against HCT 116, MDA-MB-231and FaDu cell line that showed high activity since the IC_50_ was < 100 µg/mL; while MDA-MB-231 was less sensitive and showed IC_50_ > 100 µg/mL. It has been reported that, biogenically synthesized AgNPs using lichen extracts had anticancer activity against human lung cancer cell line^35^. In their study, lower IC_50_ of 3.8 and 6.6 µg/mL were found compared with our results when they used AgNPs synthesized using *Ganoderma lucidum* and *Phellinus igniarius* respectively. Generally, anticancer activity of AgNPs prepared using green synthesis was well documented^16,35,58,88,89^. Further studies stated the IC_50_ of AgNPs synthesized using biogenic extract were ranged between 10.9 to 21.4 against HepG2, LoVo, and MDA-MB 231cell^90^ and another study showed IC_50_ ranged between 35.15–56.73 μg/mL against a LoVo cell with^16^. These differences in cytotoxicity results could be related to cell lines variations and AgNPs size, shape and concentrations. Ff-AgNPs showed more efficacy against all cell lines compared to Xa-AgNPs which might be due to the small size of the particles in Ff-AgNPs. Also, it could be related to the chemical components detected by GC–MS since higher chemicals components were identified from Ff-AgNPs compared to Xa-AgNPs. Differences in sensitivities toward different AgNPs might be due to their physiological behaviour and structural characteristics. Biogenically synthesized AgNPs may inhibit growth of cancer cell lines by various modes of actions. They may cause cellular damage in cancer cell line through the generating of ROS which leads to DNA damage by activation of Caspase-3 molecule, thus undergone apoptosis^17^. It could be concluded that, AgNPs may produce free radicals that lead cell oxidative stress, subsequently programmed cell death via caspase cascade pathway^58^.

## Conclusion

In the light of our current findings, it might be concluded that methanol extracts of lichen could be potential candidate in reducing and capping of AgNPs in the context of eco-friendly approach for extracellular synthesis in one step. The FTIR analysis showed participation of different lichen biomolecules in reducing and capping of AgNPs. The biogenic AgNPs were well-dispersed spherical of 1–145 nm which was confirmed by TEM and zeta size analysis. The eco-friendly technique presented here might be practical for AgNPs production in large-scale. Encouragingly, the biogenic AgNPs showed promising materials in antibacterial applications against *P. aeruginosa*, MRSA, VRE, and *E. coli*. Additionally, AgNPs influence on gram-negative was greater than that on gram-positive bacteria and higher cytotoxicity against FaDu and HCT 116 cell line in relation to MDA-MB-231 was found. Furthermore, substantial antibacterial and a good synergistic effect for AgNPs when associated with antibiotics might help in antibiotic-resistant microorganism treatment. Therefore, AgNPs prepared in the current study might be recommended for biomedical and pharmaceutical applications. Our study is considered as the first report that studied AgNPs fabricated using lichen types *Xanthoria parietina* and *Flavopunctelia flaventior* and approved their activity and synergistic impact with some antibiotics against pathogenic bacteria as well as cytotoxicity against three cell lines.
